# The Role of Crosstalk between Adipose Cells and Myocytes in the Pathogenesis of Sarcopenic Obesity in the Elderly

**DOI:** 10.3390/cells11213361

**Published:** 2022-10-25

**Authors:** Mauro Zamboni, Gloria Mazzali, Anna Brunelli, Tanaz Saatchi, Silvia Urbani, Anna Giani, Andrea P. Rossi, Elena Zoico, Francesco Fantin

**Affiliations:** 1 Geriatrics Division, Department of Surgery, Dentistry, Pediatric and Gynecology, Healthy Aging Center, University of Verona, 37126 Verona, Italy; 2 Geriatrics Division, Department of Medicine, University of Verona, 37126 Verona, Italy; 3 Geriatrics Division, Department of Medicine, AULSS2, Ospedale Ca’Foncello, 31100 Treviso, Italy

**Keywords:** sarcopenic obesity, adipose tissue, skeletal muscle, myokines, adipokines

## Abstract

As a result of aging, body composition changes, with a decline in muscle mass and an increase in adipose tissue (AT), which reallocates from subcutaneous to visceral depots and stores ectopically in the liver, heart and muscles. Furthermore, with aging, muscle and AT, both of which have recognized endocrine activity, become dysfunctional and contribute, in the case of positive energy balance, to the development of sarcopenic obesity (SO). SO is defined as the co-existence of excess adiposity and low muscle mass and function, and its prevalence increases with age. SO is strongly associated with greater morbidity and mortality. The pathogenesis of SO is complex and multifactorial. This review focuses mainly on the role of crosstalk between age-related dysfunctional adipose and muscle cells as one of the mechanisms leading to SO. A better understanding of this mechanisms may be useful for development of prevention strategies and treatments aimed at reducing the occurrence of SO.

## 1. Introduction

Normally, bone, muscle and fat mass grow in harmony in the body. However, this linkage may be lost as a result of aging and in several chronic diseases, when a progressive decline of muscle mass in stable weight subjects occurs, configuring a condition called sarcopenia, or when a combination of decline of muscle mass together with an increase in fat mass in overweight or obese subjects occurs, a condition called sarcopenic obesity (SO). SO has been recently defined as the co-existence of excess adiposity and low muscle mass and function. Its prevalence increases with age and has been recognized to be strongly associated with greater morbidity and mortality [1].

**Figure 1:**
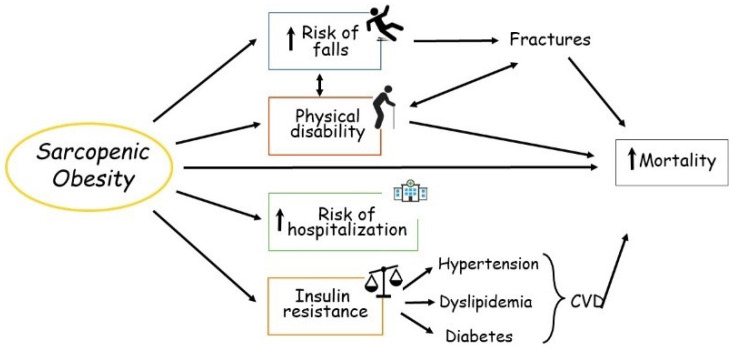
Main consequences of sarcopenic obesity in the elderly. CVD, cardiovascular disease.

Much evidence shows that the consequences of SO are clinically relevant [1,2,3]. SO has consistently been demonstrated to be a strong and independent risk factor for frailty, metabolic disorders, hospitalization and mortality in the older population (Figure 1).

Pathogenesis of SO is multi-factorial [3].

Gain in adipose tissue (WAT) and especially dysfunctional WAT may represent an independent determinant for the development of loss and dysfunction of muscle mass. In addition, a decline in muscle mass may facilitate WAT accumulation. SO is more frequently present in older adults, particularly because of the changes observed in body composition (i.e., changes in muscle mass and AT quantity and quality), which in general accompany the aging process. 

**Figure 2:**
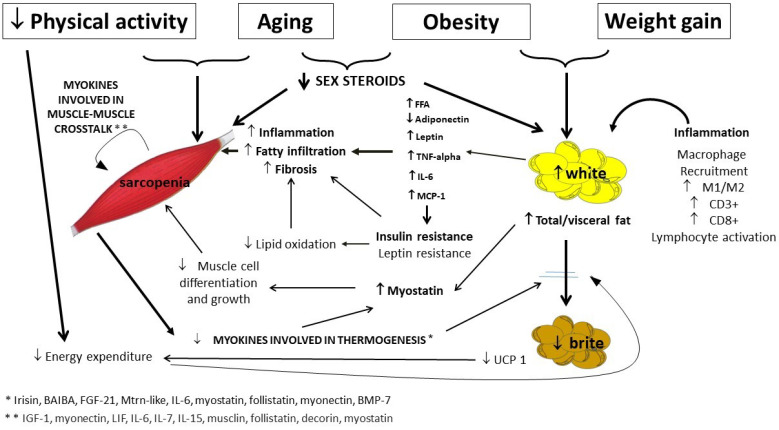
Pathogenesis of sarcopenic obesity in the elderly, with a focus on muscle and adipose tissue crosstalk. More relevant links in bold. FFA, free fatty acids; TNF-alpha, tumor necrosis factor-alpha; IL-6, Interleukin-6; MCP-1, monocyte chemoattractant protein-1; UCP-1, uncoupling protein 1.

Age-related changes in sex steroids should be also taken into account in the pathogenesis of SO in the elderly. In fact, in men as in women, age-related decline in sex steroids is strongly related to sarcopenia [4], in term of loss of muscle mass, quality and function, as well as to an increase of WAT and its redistribution (Figure 2) [5].

Both WAT and muscle have been identified as endocrine organs, which influence each other through several mechanisms [6,7]. Defects in the crosstalk between adipose cells and myocytes may be a cause of SO; an understanding of the interplay between adipose cells and myocytes may be crucial for SO prevention and/or treatment.

## 2. Adipose Tissue as Endocrine Organ 

The concept that WAT, apart from serving as an energy storage and mechanical protection, acts as an endocrine organ, representing a source of countless adipokines, proteins, metabolites, lipid molecules, non-coding RNAs and extracellular vesicles (Evs), as involved in tissue crosstalk, has been widely accepted in recent years [6]. Besides adipocytes, WAT contains stromovascular cells and immune cells acting as an integrated unit, all of which contribute to its endocrine activity [8]. Several adipocyte-derived proteins with endocrine function have been detected since the discovery of leptin many years ago [6].

WAT, distributed in different depots in the body, expresses specific features in relation to cellular composition and functions [6]. Furthermore, the anatomical location is relevant when considering that hormones produced by the visceral adipose tissue (VAT) are released into the portal system and go directly to the liver, while the systemic circulation receives the molecules produced by the subcutaneous adipose tissue (SAT) [8,9].

Moreover, WAT is a dynamic organ that modifies in response to changes in nutritional state, through modifications of its metabolism and cellularity, and subsequent shifts in adipokine secretion. In the lean state, SAT is characterized by smaller cell size and by an increased number of adipocytes, which have a different pattern of adipokine secretion compared with VAT. Weight gain causes adipocyte hypertrophy, with differences across sex, age and fat depot. Weight gain in women, at least until menopause, characterized by the enhanced accumulation of SAT, is associated with a low risk of type 2 diabetes and cardiovascular diseases [10]. In contrast, weight gain in men usually accumulates more VAT, resulting in higher metabolic risk [9].

In subjects with overweight and obesity, changes in WAT depots and cellularity, with a prevalent pro-inflammatory adipokine secretion profile, may directly contribute to the development of metabolic and cardiovascular consequences of obesity [6,9].

Adiponectin and leptin are the two most widely studied adipocyte-derived factors. Most leptin is secreted by SAT [11,12], flows into the bloodstream, passes through the blood brain barrier and arrives in areas of the brain involved in regulating hypothalamic energy balance. The link between neuroendocrine and sympathetic control of WAT endocrine function, and the existence of negative feedback between the brain and WAT, have been widely studied [13]. In the last few decades, several studies have shown the association between leptin and cardiovascular diseases [14].

Adiponectin is an adipokine with described anti-atherogenic, anti-inflammatory and insulin-sensitizing properties [15]. Obesity is associated with reduced adiponectin expression in VAT, and adiponectin levels have been shown to negatively correlate with the amount of VAT [16], suggesting that the obesity-associated decline in adiponectin could contribute to the detrimental effects of excessive VAT accumulation on whole body metabolism [17].

VAT expansion also triggers other proinflammatory cytokine expressions as well as the recruitment of immune cells [18]. Expansion of VAT is accompanied by increased interleukin 6 (IL-6) and tumor necrosis factor (TNF) secretion, which lead to a crown-like structure formation, and augmented hypoxia-inducible factor 1α expression to promote angiogenesis, which contributes to local and systemic inflammation [19,20,21]. 

Moreover, great attention has recently been focused on WAT contribution in inter-organ communication, not only by producing signaling mediators but also by converting or degrading signaling mediators from other organs (“signal metabolism” and “signal catabolism”) [6]. Increasing and specific interest has been given to Evs (carrying protein, lipids, small coding and noncoding RNAs), which are now considered an eminent way of communication between WAT and other organs, as well as between different cell populations within WAT itself [6].

## 3. Adipose Tissue Changes across Aging 

With aging, quantity, distribution, and function of WAT changes. Fat mass increases and reaches its peak at about 65–75 years for men, and later for women [22]. The increase in fat mass is independent from changes in body weight, and this is due to the concurrent decline in muscle mass: so-called sarcopenia [23]. Fat storage is progressively redistributed from the body’s periphery (i.e., loss of SAT, in particular, gluteo-femoral SAT) to the abdomen (i.e., increase of VAT), and such abdominal fat accumulation is independent of weight gain [24,25]. It has been shown that involuntary SAT loss, in the absence of a negative energy balance, is associated with triglyceride (TG) spillover, which determines ectopic deposition of TG in muscle, liver, bone marrow and heart, contributing to the dysfunction of these organs [24,25].

With aging, WAT also becomes dysfunctional, showing an increased profile of pro-inflammatory adipokines produced by adipose cells, greater infiltration of inflammatory cells in WAT, and preadipocytes and adipocyte incompetence, together leading to inflammaging [26].

In general, serum levels of the majority of adipokines are higher in older than in younger individuals [24]. The relationship between aging and the endocrine function of WAT is complex to study in humans. As aging is associated with changes in fat mass and its distribution, as well as with high prevalence of metabolic syndrome, insulin resistance and obesity, the effect of aging itself is difficult to isolate. Indeed, age-related VAT increase, obesity and metabolic syndrome are all factors that can induce an increase in inflammatory and a decrease in anti-inflammatory adipokine production.

Older subjects show higher leptin levels [27], whose activity seems to be reduced, thereby determining a phenomenon called leptin resistance, a phenomenon that is not completely understood in humans [28]. 

It has been determined that the amount of serum adiponectin rises as humans age [29], and higher levels of adiponectin have been found in centenarians [30]. Although the beneficial metabolic and anti-inflammatory effects of adiponectin have been confirmed by some scientific studies, adiponectin’s role in the elderly is still controversial [30,31]. Indeed, a significant positive relationship was found between adiponectin and risk of incident disability and all-cause mortality among the subjects of the Health ABC Study [32], but this relationship was not significant after adjusting for weight loss and physical performance at baseline.

In an in vitro model of chronological aging of adipocytes, we and other researchers observed that adipocyte secretion of proinflammatory cytokines, such as interleukin-6 and monocyte chemoattractant protein-1, was significantly higher in older than younger adipocytes [33,34,35]. We also found that in vitro aged adipocytes accumulate ROS, increase mRNA expression of key proteins related to the remodeling of the extracellular matrix and increase p53, p21 and p16 expression, compared to younger cells [34,35].

Moreover, dysfunction of WAT is also characterized by increased oxidative stress (OS), mitochondrial dysfunction, reduction in vascularization and hypoxia [36]. The age-related deregulation of WAT can initiate inflammatory cycles with monocyte recruitment and activation of macrophages. The ratio between pro-inflammatory M1 and anti-inflammatory M2 macrophages increases with aging [37,38], as well accumulation of CD3+ T and CD8+ T cells and activation of T and B lymphocytes [39,40]. Factors secreted by activated macrophages induce the release of fatty acids from adipocytes that inhibit differentiation of pre-adipocytes and cause de-differentiation of mesenchymal progenitors into mesenchymal adipocyte-like default cells [41]. These processes induce lipotoxicity both in WAT and in other organs, cause cellular stress responses, promote release of inflammatory cytokines, block adipogenesis and determine further release of lipotoxic fatty acid [42]. WAT in the elderly is also characterized by reduction of adipocyte size and increase of tissue fibrosis [43]. 

That adipocyte size is affected by aging was clearly shown by Donato et al., who observed significantly lower adipocyte areas from WAT in older than younger mice [44] and by the fact that a high fat diet was able to increase adipocyte diameter in young rats but not in old rats [45]. Furthermore, age-related decline of adipocyte capacity to stock TG has been shown to be related to fat infiltration inside the muscles, as well as to a deposition in muscles of toxic lipids such as ceramides [45]. Fibrosis, characterized by an increase in connective fiber content, may be due to an up-regulation of collagen protein [46]. The OS that occurs in WAT during aging causes oxidative damage to lipids, proteins and DNA. Finally, senescent cells are over-represented in aged WAT, providing a source of many pro-inflammatory cytokines and chemokines, impairing the production of extracellular matrix modifying proteases and further promoting the production of ROS [47,48].

## 4. Muscle as Endocrine Organ

Muscle has also been identified as having a secretory/endocrine function [7]. Cytokines and other peptides produced, expressed and released by muscle cells are called myokines. Proteomics analyses identified over 650 proteins and peptides produced by muscle cells, yet their precise biological role has been characterized only in a minority of cases [49]. Myokines act in an autocrine, paracrine or endocrine way. In fact, some myokines exert their effect on muscle itself, taking part in muscle hypertrophy and myogenesis, while other myokines are involved in the regulation of energy metabolism. There is now solid evidence that skeletal muscle, through the production of myokines, communicates with other key organs and regulates lipid mobilization from adipose tissue, liver endogenous glucose production, insulin secretion and thermogenesis [50]. 

Many myokines are produced in response to the contraction of muscle fibers and may indeed mediate protective effects of physical exercise and counteract the pathological consequences of a sedentary lifestyle.

Several myokines act precisely within skeletal muscle itself and are involved in muscle cell proliferation, differentiation, and regeneration [51,52]; others are involved in mediating energy supply during exercise (Table 1). 

It is now believed that every stage of the myogenic process involves regulation by myokines, many of which contribute to myogenic regulation at different stages, from satellite cell proliferation to differentiation and cell survival [49,53].

The role of IGF-1 and IGF-2 as endocrine modulators of myogenesis has been extensively studied [54], as they seem indispensable for the initiation of differentiation [55]; IGF-1 leads to muscle hypertrophy by activating satellite cells and possibly inhibiting autophagy [56]. Impairment of IGF-1 signaling has been described in chronic disorders, and as such, represents a possible mechanism in muscle atrophy led by altered protein synthesis, autophagy, and impaired muscle regeneration [57].

Furthermore, IL-7 plays an essential role in myogenesis and may influence the differentiation of satellite cells into fully developed skeletal muscle cells [58].

On the other hand, TGF-beta has shown to be a strong inhibitor of myogenic differentiation in vitro [59]. Myostatin, the first discovered myokine, is a member of the TGF-beta superfamily and plays a key role in muscle growth and differentiation, by controlling the proliferation of myoblasts (as a major negative regulator of skeletal muscle growth) and suppressing satellite cell activation and myoblast proliferation [60,61]; it is known that the deletion of the myostatin gene causes massive muscle hypertrophy in animals [62,63].

More recently, follistatin and decorin have been identified as potent inducers of muscle hypertrophy with an anti-myostatin function [64]; in particular, decorin acts in an auto/paracrine manner as a direct antagonist of myostatin [65]. Although myokine IL-6 is mainly known for its role in the regulation of lipid and glucose metabolism, it has also been shown that it has an anabolic effect in the processes of myogenesis [66]. Leukemia inhibitory factor (LIF) also exerts an autocrine/paracrine action [67], and it has proven to be crucial for satellite cell proliferation and survival [67].

Apart from the regulatory effects on myogenesis, many myokines act on metabolic pathways in the modulation of energy metabolism. IL-6 [50] and brain-derived neurotrophic factor (BDNF) [68] are involved in activating fat oxidation in muscle cells. Indeed, IL-6 signaling within muscle cells appears to affect both glucose uptake and fat oxidation, and its role in GLUT4 translocation has been described [69]. In addition, several studies described an increase in intramyocellular and whole-body fatty acid oxidation in response to myokine IL-6 [69,70]. BDNF affects myogenesis through activation of satellite cells [71], especially in response to muscle injury; it has also been suggested as a regulator of neuromuscular function during the aging process, with possible implications in sarcopenia and SO [72]. Indeed, low levels of BDNF are described in subjects with obesity and T2D [73]. 

**Table 1:** Selected myokine functions.

Function	Myokine	Aging	References
Myogenesis and muscle hypertrophy	myostatin LIF IL-6 IL-7 IL-15 musclin follistatin decorin myonectin IGF-1 musclin	⇑ ? ⇑ ⇓ ⇓ ? ⇓ ⇓ ⇓ ⇓ ?	[61,74] [67] [66,75] [58] [76] [77] [64,78] [79,80] [81] [56] [82]
Muscle-cell FFA oxidation	IL-6 BDNF irisin myonectin	⇑ ⇓ ⇓ ⇓	[69,83] [68] [84] [81]
Insulin sensitivity	IL-6 IL-15 SPARC LIF BMP-7 mitsugumin 53	⇑ ⇓ ⇓ ? ⇓ ?	[7,69] [85,86] [87] [88] [89] [90]
Osteogenesis	IGF-1 decorin IL-6	⇓ ⇓ ⇑	[91] [92] [93]
Browning of WAT	Irisin IL-6 meteorin-like FGF-21 BAIBA follistatin myonectin BMP-7	⇓ ⇑ ? ⇓ ⇓ ⇓ ? ⇓	[84] [94] [95] [96] [97,98] [99] [100] [101]
Lipolysis	IL-6 FGF-21 ANGPTL-4	⇑ ⇓ ?	[7] [102] [103]
Muscle innervation	BDNF FGFBP-1	⇓ ?	[104] [105]
Muscle angiogenesis	IL-8 VEGF-A	⇓ ?	[76] [106,107]

LIF: leukemia inhibitory factor; IL-6: interleukin-6; IL-7: interleukin-7; IL-15: interleukin-15; BDNF: brain-derived neurotrophic factor; IGF-1: insulin-like growth factor-1; SPARC, secreted protein acidic and rich in cysteine; BMP-7, bone morphogenetic protein-7; FGF-21: fibroblast growth factor 21; BAIBA: β-aminoisobutyric acid; CNTFR-A: ciliary neurotrophic factor receptor-A; ANGPTL-4, angiopoietin-like protein 4; FGFBP-1, fibroblast growth factor binding protein 1; VEGF-A: vascular endothelial growth factor-A.

## 5. Muscle Mass Changes across Aging 

Aging-related decline in skeletal muscle structure (quantity and quality) and function, known as sarcopenia, occurs as a result of aging. This process takes place slowly but represents a critical and significant event during the aging process. 

After the age of 50, muscle mass declines yearly in men and in women, with reduced muscle fiber number and size, mainly due to a progressive loss of motoneurons. 

Muscle quality significantly changes with aging, and this process occurs earlier than the reduction in muscle mass. The muscle’s architecture is modified, type II fibers decline, and vasculature is reduced. Fat deposition inside muscles, called myosteatosis and characterized by both inter- and intra-muscular fat, rises significantly with aging together with fibrosis affecting insulin sensitivity as well as peak muscle force generation, leading to impaired mobility and metabolic dysfunction in older adults [108,109]. 

Modification of muscle proteins and loss of coordinated control between contractile, mitochondrial and sarcoplasmic reticulum protein occur, with mitochondria alterations. Finally, progressive motoneuron loss occurs, and this is not adequately compensated by reinnervation of muscle fibers by the remaining motoneurons [108].

Aging-related muscle changes are also due to the secretion of myokines. The contents of various myokines, such as interleukin IL-6, irisin, myostatin, brain-derived neurotrophic factor (BDNF) and apelin change correspondingly with increasing age [110]. During aging, an increase in the muscle cells’ expression of pro-inflammatory cytokines (IL-6, IL-1β and TNF-α) is observed, leading to skeletal muscle atrophy [108].

Absolute serum irisin concentrations are significantly higher in the young compared to older adults. With increasing age and the occurrence of muscle atrophy in aged mice and older humans, the level of circulating irisin decreases [111].

Serum levels of BDNF myokine, widely expressed in different cell types and essential in regulating cardiomyocyte contraction, also decrease with aging [112]. 

Apelin, through the activation of AMPK signaling, has been shown to be an important promoter of mitochondrial biogenesis and muscle cell regeneration. Apelin production is induced by muscle contraction and is reduced with aging [113].

Myostatin is the most famous myokine in the muscle field; besides its well-established role in muscle wasting, there are gaps in the evidence. For instance, the relationship between serum myostatin and skeletal muscle mass in humans remains controversial [114]. Some authors observed an inverse relationship between age-related muscle loss and serum myostatin levels in the frail elderly [115], while skeletal muscle atrophy associated with lower myostatin levels has also been observed [116].

## 6. Muscle–Adipose Tissue Crosstalk: Role of Myokines

There is strong evidence, from proteomics studies using in vitro and in vivo models, that many myokines are key endocrine mediators in glucose and lipid metabolism, especially in response to exercise, through crosstalk with other tissues, including WAT. IL-6 is known to increase lipolysis and fatty acid release from WAT [117]; it has been observed that exercise training leading to reduction in VAT was avoided by IL-6 receptor blockade with tocilizumab (IL-6 receptor antibody) [118].

Irisin is one of the myokines of greatest interest, produced in response to muscle contraction and secreted after cleavage of the intracellular form FNDC5 [84]; irisin influences energy homeostasis, and it is a key regulator in adipocyte metabolism, since it is thought to be a bridge between exercise and metabolic homeostasis. Irisin has been proposed as the main mediator in WAT browning and an activator of thermogenesis, which promotes an increase in energy expenditure, thus preventing fat gain [119]. Irisin also improves the structure and strength of cortical bone, as a key player in muscle–bone crosstalk [120,121]. 

Myonectin has also been shown to act on WAT; as with irisin, it is produced in response to muscle contraction and appears to be involved in the regulation of FFA uptake in adipocytes in vitro [81]. Interestingly, myonectin transcription in muscle cells can be up-regulated with the addition of FFA and glucose, which suggests a role in muscle–adipose tissue crosstalk that informs tissues of nutrient status and promotes nutrient uptake and storage [81,122].

Evidence suggests a unique picture for the action of FGF-21 in muscle and adipose tissue crosstalk [102]; FGF-21 has also been shown to be secreted by muscle cells, in addition to the liver and WAT, which contributes to reducing blood glucose and plasma TG levels as well as increasing insulin sensitivity [123]. In fact, FGF21 produced by muscle cells seems to have an interesting effect on adipocytes, modulating their gene expression, for example, by increasing the expression of adiponectin [124]. Kim et al. [125] demonstrated that FGF21 deficiency exacerbated obesity-induced inflammation and atrophic responses in the skeletal muscle of obese mice. Collectively, these data suggest that FGF21 could protect the body against obesity and insulin resistance [123].

Apelin was, until recently, described as an adipokine [126] whose expression and circulating levels were increased in obesity [127]; further evidence showed that apelin is also produced by myocytes during muscle contraction [128]. Available evidence indicates that it has beneficial effects on glucose and lipid metabolism both in WAT and skeletal muscle [129,130,131,132]. 

Alongside its role in promoting muscle trophism, improvement of glucose tolerance has also been described for decorin; decorin-knockout mice showed higher leptin levels and impaired glucose tolerance [133].

Recent evidence suggests that several myokines may induce browning of WAT, thus enhancing global energy expenditure [134]. In fact, exercise-induced circulating factors (“exerkines”) are able to modulate activation of brown adipose tissue (BAT) and browning of WAT by activating uncoupling protein 1 (UCP1) [135]. Exercise training as well as cold exposure and dietary components are associated with the enhanced accumulation of metabolically active beige adipocytes and BAT activation in adult humans [136,137]. The expression of UCP1 is modulated by PGC-1α, which is dependent on several myokines: irisin, βaminoisobutyric acid (BAIBA), myostatin, follistatin, decorin, meteorin-like (Mtrn-like), IL-6 and lactate [134]. The regulation of the PI3K-AKT pathway and the expression of UCP1 in BAT is upregulated by irisin [135]. A role of FGF-21 in the browning of WAT has also been hypothesized [94,96].

Other molecules potentially implicated in WAT browning and energy expenditure regulation include BAIBA and meteorin-like [95]. BAIBA is a small aminoacidic metabolite derived from valine and thyamine catabolism and is released by contracting myocytes [97]; its role in the beiging of WAT has been described by Roberts et al. [97], and it has also been hypothesized as an actor in suppressing inflammation in skeletal muscle and WAT, as well as in boosting insulin sensitivity [97,138,139]. Similar to many other myokines, its levels are reduced with aging [140,141]. BAIBA involves specific PPARα-dependent mechanisms and exhibits an increased BAT-specific gene expression (Pgc1a, Ucp1, Cidea and Cytc) in both mice and humans, as well as increased mitochondrial activity [134]. The Mtrn-like glial cell differentiation regulator is a novel protein secreted by muscle cells that increases after training and promotes mitochondrial gene programs in WAT and energy expenditure by increasing the brown/beige shift of adipose cells [95]. The browning effect of Mtrn-like shared with the better known irisin, however, seems to be mediated by an indirect mechanism, with the involvement of immune cells in the muscle and WAT crosstalk [95]. Moreover, the myogenic effects of Mtrn-like seem to be mediated by an anti-inflammatory response triggered by macrophage activation [142]. The release into circulation of Mtrn-like indirectly leads to an increased expression of UCP1, via an eosinophil-dependent mechanism and the eosinophil-specific chemokines IL-4 and IL-13, and promotes the activation of WAT macrophages, which produce catecholamines and ultimately activate a pro-thermogenic program [49,134].

## 7. Adipose Tissue Muscle Crosstalk: Role of Adipokines

The first evidence of a link between WAT and muscle mass arises from the fact that muscle alterations in terms of quantity and quality are observed in individuals with both WAT deficiency [143] and excess.

WAT excess or dysfunction may be related to muscle damage through the excessive FFA production by hypertrophic adipocytes that may accumulate in and between muscle fibers ectopically. This determines mitochondrial dysfunction, increased ROS production during FFA oxidation and accumulation of reactive lipids in skeletal muscle and leads to lipotoxicity, a process that contributes substantially to the pathophysiology of insulin resistance, sarcopenia and SO [144]. 

Furthermore, several adipokines have been shown to produce positive or negative effects on skeletal muscle [145,146]. 

Hypertrophic adipocytes increase the secretion of proinflammatory adipokines, determining insulin resistance and thus muscle protein catabolism and protein synthesis inhibition [8,147].

Leptin is one of the most abundant circulating adipokines released by SAT. It induces myocyte cell proliferation and some myogenic factors, since it can also suppress myostatin as a negative regulator of muscle growth and some atrophy markers such as MuRF1, which promotes muscle growth. Leptin activates the Akt/mechanistic target of the rapamycin (mTOR) signaling cascade [148,149]. C2C12 derived myotubes’ exposure to leptin has been shown to increase protein synthesis, decrease degradation and increase myoblast proliferation [150].

The role of leptin in muscle mass has been recently confirmed in a study conducted in fat-free lipodystrophic mice with decreased muscle mass and strength, in which a full rescue of muscle mass, in term of quantity and function, was observed after replacement of just ∼10% of normal WAT, and in which this effect was shown to be independently due to leptin and separable from the reversal of systemic metabolic derangement [151].

However, in the case of obesity, aging and chronic disease, hyperleptinemia induces leptin resistance, which limits muscle FFA oxidation and precludes any of the above-mentioned positive effects of leptin on muscle. 

Leptin activates white adipose cell differentiation towards a BAT-like phenotype through the activation of sympathetic nerve activity. Furthermore, leptin and insulin act synergistically on distinct POMC neuronal subsets to promote WAT browning. Interestingly, leptin has different effects in skeletal muscle and in SAT. In the former, it increases Fndc5 gene expression levels and stimulates irisin-induced muscle growth, while in the latter, it downregulates the transcript levels of Fndc5 [134]. Adipocyte-derived FGF-21 activates the thermogenic gene expression, mediated by central (via sympathetic activation) and local (via induction of the PGC-1α protein) mechanisms. Anti-inflammatory M2 macrophages, whose proliferation is promoted by adiponectin, have recently been proposed as an important source of norepinephrine, a hormone involved in browning and thermogenesis in BAT [134].

Adiponectin is another peptide secreted by adipocytes with known anti-inflammatory and insulin sensitizer properties. Adiponectin has also been found to be expressed by skeletal muscle cells [152]. Adiponectin increases FFA oxidation and glucose uptake in skeletal muscle and inhibits hepatic gluconeogenesis. Furthermore, high intensity aerobic physical activity has been shown to increase plasma levels of adiponectin [110], and upregulation of adiponectin receptors in skeletal muscle of severely obese subjects in response to endurance training has been observed. Some evidence of the effect of resistin and chemerin on muscle cells has been observed in vitro: chronic incubation of resistin in skeletal muscle cells has been found to decrease fatty acid uptake and metabolism as well as to reduce basal and insulin-stimulated glucose uptake, oxidation, and glycogen synthesis, while incubation of skeletal muscle cells with chemerin promotes proliferation and suppresses differentiation of muscle cells through ERK1/2 and mTOR signaling pathways [145,153]. Chemerin, whose gene expression has been shown to be altered in WAT and skeletal muscle of obese/diabetic mice, worsens insulin sensitivity in myocytes and adipocytes [154]. 

BAT is also able to secrete different factors, called batokines, such as prostaglandin, endothelin, IL-6, fibroblast growth factor-21, myostatin and CXCL14, which contribute to fat browning [155]. Preadipocytes from myostatin-deficient mice have exhibited an increased propensity to differentiate into brown fat cells, which secrete CXCL14, leading to adaptive thermogenesis via M2 macrophage recruitment, enhancing BAT activation as well as the browning of WAT [134].

## 8. Adipomyokines and MicroRNA in the Crosstalk between AT and Muscle

Overlap between myokines and adipokines exists; in fact, several cytokines, called adipomyokines, secreted from skeletal muscle cells, are also secreted by adipocytes [110,156], confirming the existence of a strong interplay between muscle and WAT. Adipomyokines show a variety of actions; a general overview is summarized in Table 2. Myostatin, known as an inhibitor of myocyte differentiation and proliferation, is one of the best characterized adipomyokines.

Myostatin is upregulated in animal models of obesity, and elevated myostatin levels have been observed in obese subjects [157,158]. By collecting muscle biopsies in extremely obese women, a strong correlation has been found between the gene expression of myostatin and BMI and insulin-resistance [159]. Apart from its effects on muscle trophism and metabolism [160], myostatin has also shown to be a positive regulator of adipogenesis [161]. Across aging, augmented levels of myostatin may lead to the reduction of the age-related muscle mass [115]; this effect could also be accentuated by the possible role of myostatin in the inhibition of irisin synthesis, which contributes to decline in muscle mass as well as to rise of fat mass, and ultimately leads to SO [162]. Myostatin is negatively related to the expression of key brown (Pgc1a, Ucp1, Prdm16, Cidea and Dio2) and beige (Tmem26 and Cd137) WAT-specific genes [163]. Loss of myostatin leads to decreased miR-34a expression, which subsequently promotes Fndc5 expression, thereby increasing thermogenic gene expression and browning in WAT.

IL-15 is an exercise-modulated adipomyokine, with documented anabolic effects on muscle, for example, by reducing protein degradation [164] and regulatory effects on muscle oxidative metabolism, and in particular by increasing FFA oxidation and mitochondrial density [165,166] as well as reduction of lipogenesis and gluconeogenesis [167]. IL-15 can reduce VAT in mice and humans [164,168,169,170]; this effect could be mediated by IL-15 effects on adipocytes, which includes proliferation rate reduction and apoptosis [171]. Moreover, IL-15 inhibits the accumulation of lipids in preadipocytes and stimulates the secretion of adiponectin, which indirectly reduces WAT mass [170]. Evidence suggests that IL-15 is involved in reciprocal interplay between muscle and WAT, which provides beneficial effects in glucose and lipid metabolism. 

Micro RNAs can be also involved in the crosstalk between WAT and muscle mass. MiRNAs expressed in skeletal muscles are defined as myomiRs; a role in myocyte differentiation and proliferation has been described for and includes miR-133b, miR-133a, miR-208a, miR-208b and miR-486 [172,173]. MiR27 mediates the communication between AT and skeletal muscle [174]. Administration of a miR-33a mimetic to primary duck myoblasts reduced proliferation while its inhibition led to its augmentation [175]. 

**Table 2:** Selected adipomyokines in muscle and adipose tissue crosstalk.

Adipomyokine	Effects–Skeletal Muscle	Effects—Adipose Tissue	Aging	References
IL-6	+muscle hypertrophy +glucose uptake +glycogenolysis, lipolysis	+lipolysis +free fatty acid (FFA) oxidation browning of WAT	⇑	[7,69]
Irisin	+glucose uptake +muscle trophism	+lipolysis browning of WAT	⇓	[176,177]
IL-15	+glucose uptake +mitochondrial activity	−lipid accumulation +adiponectin secretion	⇓	[169,171,178]
BAIBA	+mitochondrial FFA oxidation +insulin sensitivity	+mitochondrial FFA oxidation	⇓	[97,139]
Meteorin-like	+energy expenditure +glucose tolerance	browning of WAT	?	[95]
LIF	+muscle hypertrophy +satellite cell proliferation regeneration after muscle damage	+adipocyte differentiation	⇓	[67]
Myostatin	- muscle hypertrophy	+adipogenesis	⇑	[60,161,179,180]
Apelin	improves muscle metabolism	glucose uptake −lipid storage	⇓	[129,130,131,132]
ANGPTL4	+FFA oxidation	+lipolysis	?	[103,181]
FGF-21	+thermogenesis	+glucose uptake	⇓	[182,183]
Follistatin-like 1	+endothelial cells function and survival		?	[184,185]
IL-8	+insulin resistance	+insulin resistance	⇓	[186,187]
MCP-1	−glucose uptake		?	[187]
PEDF	+insulin resistance +ectopic lipid deposition	+insulin resistance +pro-inflammatory pathway	?	[188,189]

IL-6: nterleukin-6; IL-15: interleukin-15; BAIBA: β-aminoisobutyric acid; LIF: leukemia inhibitory factor; ANGPTL-4, angiopoietin-like protein 4; FGF-21: fibroblast growth factor-21; IL-8, interleukin-8; MCP-1, monocyte chemoattractant protein-1; PEDF, pigment epithelium-derived factor; "−": decreased; "+": increased.

## 9. Conclusions 

With aging, loss of muscle mass and gain in fat occur and contribute, in the presence of a positive energy balance, to the development of SO [1], a condition frequently observed in the elderly. SO has been clearly recognized as a clinical condition linked to worse outcomes than obesity itself [1,2,190]. Hence, prevention and treatment of SO are mandatory. Crosstalk between muscle cells and adipose cells is one of many mechanisms that lead to SO. However, prevention strategies may help to reduce the occurrence of SO by correcting crosstalk between muscle and adipose cells.

There is some evidence that the release of some myokines by the skeletal muscle is increased by physical activity, across all age groups [191,192]. Yet the evidence currently available paints a complex picture, and there are still some gaps concerning the types and intensity of physical exercise required [193].

Some studies have already investigated the influence of nutrition on myokine production [194]. A role in the modulation of WAT and muscle cell function has been hypothesized for caloric restriction, dietary supplementation of polyphenols, prebiotics or probiotics, and 3-n PUFA [194]. Prevention of weight gain with aging as well as the promotion of an active lifestyle may be a strategy for preserving WAT function and muscle mass and to improve the interplay between muscle mass and WAT.
